# Being in a Romantic Relationship Is Associated with Reduced Gray Matter Density in Striatum and Increased Subjective Happiness

**DOI:** 10.3389/fpsyg.2016.01763

**Published:** 2016-11-14

**Authors:** Hiroaki Kawamichi, Sho K. Sugawara, Yuki H. Hamano, Kai Makita, Masahiro Matsunaga, Hiroki C. Tanabe, Yuichi Ogino, Shigeru Saito, Norihiro Sadato

**Affiliations:** ^1^Division of Cerebral Integration, Department of Cerebral Research, National Institute for Physiological SciencesOkazaki, Japan; ^2^School of Medicine, Faculty of Medicine, Gunma UniversityMaebashi, Japan; ^3^Graduate School of Human Health Sciences, Tokyo Metropolitan UniversityTokyo, Japan; ^4^Department of Physiological Sciences, SOKENDAI (The Graduate University for Advanced Studies)Hayama, Japan; ^5^Department of Health and Psychosocial Medicine, School of Medicine, Aichi Medical UniversityNagakute, Japan; ^6^Department of Social and Human Environment, Graduate School of Environmental Studies, Nagoya UniversityNagoya, Japan; ^7^Department of Anesthesiology, Graduate School of Medicine, Gunma UniversityMaebashi, Japan

**Keywords:** gray matter density, romantic relationship, striatum, subjective happiness, voxel-based morphometry

## Abstract

Romantic relationship, a widespread feature of human society, is one of the most influential factors in daily life. Although stimuli related to romantic love or being in a romantic relationship commonly result in enhancement of activation or functional connectivity of the reward system, including the striatum, the structure underlying romantic relationship-related regions remain unclear. Because individual experiences can alter gray matter within the adult human brain, we hypothesized that romantic relationship is associated with structural differences in the striatum related to the positive subjective experience of being in a romantic relationship. Because intimate romantic relationships contribute to perceived subjective happiness, this subjective enhancement of happiness might be accompanied by the experience of positive events related to being in a romantic relationship. To test this hypothesis and elucidate the structure involved, we compared subjective happiness, an indirect measure of the existence of positive experiences caused by being in a romantic relationship, of participants with or without romantic partners (*N* = 68). Furthermore, we also conducted a voxel-based morphometry study of the effects of being in a romantic relationship (*N* = 113). Being in a romantic relationship was associated with greater subjective happiness and reduced gray matter density within the right dorsal striatum. These results suggest that being in a romantic relationship enhances perceived subjective happiness via positive experiences. Furthermore, the observed reduction in gray matter density in the right dorsal striatum may reflect an increase in saliency of social reward within a romantic relationship. Thus, being in a romantic relationship is associated with positive experiences and a reduction of gray matter density in the right dorsal striatum, representing a modulation of social reward.

## Introduction

Romantic love, which is essentially a human universal, is widespread across cultures throughout the world (Jankowiak and Fischer, 1992) and is presumed to be experienced by the vast majority of people. Romantic love serves as an evolutionary device that encourages couples to stay together and attend to their helpless infants (Gonzaga and Haselton, 2008). Additionally, higher levels of commitment in romantic relationships are associated with a tendency to underrate attractive alternatives (Johnson and Rusbult, 1989; Maner et al., 2009); thus, romantic love functions to maintain relationships and diminish the desire to search for alternative mates. In this sense, romantic love is an essential aspect of well-being and positive experience in human society.

Romantic love can be regarded as a deep and meaningful affective state. Romantic love begins as passionate love, which is a state of total absorption between two individuals (Berscheid and Walster, 1978). As the concept of “you and me” transitions to the concept of “us,” romantic love has a positive impact on one’s existence (Aron et al., 1995). Furthermore, we can become emotionally dependent on a partner for happiness within an intimate relationship (Hazan and Shaver, 1987). Strong romantic relationships are accompanied by subjective happiness (Diener and Seligman, 2002), which leads to improvements in mental health (Glenn, 1975; Gove et al., 1983). Possibly due to the positive consequences of being in a romantic relationship, by early adulthood, time with romantic partners increases at the expense of involvement with friends (Reis et al., 1993). In this sense, being in a romantic relationship is one of most positive influential experiences of human adulthood.

In terms of neural underpinnings, romantic love has a specific neuropsychological signature, including the release of hormones such as dopamine (Takahashi et al., 2015). Dopamine tends to be focused on the nucleus accumbens of the ventral striatum through pair bonding (Aragona et al., 2006). Photographic or video images of a romantic partner activate the dorsal striatum, another target of dopaminergic projections (Bartels and Zeki, 2000, 2004; Aron et al., 2005; Xu et al., 2011; Acevedo et al., 2012). The dorsal striatal activation associated with viewing a beloved person is enhanced by attention (Langeslag et al., 2014). Furthermore, the mere presence of a romantic partner suppresses pain-related processes (Coan et al., 2006; Master et al., 2009; Eisenberger et al., 2011), concomitant with activation of the reward system including the dorsal and ventral striatum (Younger et al., 2010). In addition to the changes observed when participants perceived stimuli related to their romantic partners, resting state fMRI of persons in a romantic relationship revealed increased functional connectivity within the reward network, including the striatum, in comparison with persons whose relationships had ended or who had never been in love (Song et al., 2015). Thus, the striatal component of the reward system represents romantic love or relationship, indicating that the striatum plays a pivotal role in this phenomenon.

However, it remains unclear how romantic love or relationship is relate to human neural systems, i.e., we do not yet know which neural structure beyond the response of the striatum (i.e., striatal structure) are related to romantic love or relationship. Individual differences in adult brain structure are a rich source of information about the variability of a huge range of behaviors (Kanai and Rees, 2011). Furthermore, individual experiences can cause alterations in the gray matter of the adult brain, accompanied by synaptic remodeling (Zatorre et al., 2012). Because romantic love is one of most influential positive experiences for human beings, being in a romantic relationship might be accompanied by changes in the underlying neural structures. In terms of structural modulation, the striatum is tuned to respond to reward with subsequent influence on approach behavior (Langen et al., 2011). As with stimuli related to a romantic partner, viewing of sexual stimuli activates the reward system (Redoute et al., 2000). Furthermore, a history of time spent consuming visual sexual stimuli reduces gray matter volume in the right caudate (Kuhn and Gallinat, 2014). Other kinds of stimuli related to approach behaviors have similar effects. Patients with cocaine dependence or alcoholism exhibit reduced gray matter density in both the dorsal (Barros-Loscertales et al., 2011) and ventral striatum (Makris et al., 2008). The reduction in gray matter density in the dorsal striatum of cocaine-dependent patients may be associated with an enhanced response to reward, accompanied by deficits in inhibitory control (Barros-Loscertales et al., 2006). Similar to cocaine-dependent patients, person in early-stage romantic love exhibit reduced cognitive control (van Steenbergen et al., 2014), thus addiction and romantic relationship might share common striatal structures at least some extent. Based on these observations, we hypothesized that the striatal gray matter density should be reduced in people in romantic relationships through positive experiences.

To test this hypothesis, we measured the subjective happiness of two groups of participants, being in (in-relationship) and not being in (no-relationship) romantic relationship, to investigate the existence of positive experiences mediated by involvement of a romantic relationship. Based on the rewarding nature of romantic love (Bartels and Zeki, 2000; Aron et al., 2005), humans might have positive experiences at a higher frequency than negative experiences during a long period of time spent with a romantic partner. These positive experiences related to romantic love should enhance subjective happiness, especially in people involved in happy/functional relationships (Proulx et al., 2007). In this sense, subjective happiness is a suitable indirect measure of the existence of subjects’ positive experience related to being in a romantic relationship, even though it might be an inappropriate indirect measure of the amount of positive experience. In terms of subjective happiness rating, we expected that participants with a romantic partner would report higher subjective happiness. Next, we conducted voxel-based morphometry (VBM) study to investigate structural differences between the in-relationship and no-relationship groups. We expected to observe reduced gray matter density in the striatum of the in-relationship group. As individuals in early-stage romantic couples perceive their partners as rewarding (Burkett and Young, 2012), we selected people in early-stage romantic relationship as participants.

## Materials and Methods

Data are presented as means ± standard error of the mean (SEM) unless otherwise indicated.

### Participants

Participants were divided into two groups (in-relationship and no-relationship). The in-relationship group comprised 56 healthy volunteers (32 males and 24 females; age = 21.8 ± 0.3 years) with a reported duration of “being in a romantic relationship” of 17.0 ± 2.0 months [i.e., early-stage romantic relationships, as in a previous study (Takahashi et al., 2015)]. The no-relationship group comprised 57 healthy volunteers (32 males and 25 females; age = 21.0 ± 0.2 years). All participants were undergraduate or graduate university students. There was no significant difference in age between the two groups, as determined by *t*-test [*t*(111) = 1.84, *p* = 0.07].

In addition, we required participants to complete the subjective happiness scale (SHS, described later) immediately after the magnetic resonance imaging (MRI) measurements. Due to time constraints in the experiment (we were conducting other fMRI experiments at that time), we could not measure SHS of 45 participants. Therefore, we administered the SHS to a total of 68 participants [in-relationship group: 36 participants (20 males and 16 females; age = 21.7 ± 0.4 years); no-relationship group: 32 participants (16 males and 16 females; age = 20.9 ± 0.3 years)]. There was no significant difference in age between the two subsets, as determined by *t*-test [*t*(66) = 1.75, *p* = 0.08].

Participants were provided with monetary compensation. The protocol was approved by the ethical committee of the National Institute for Physiological Sciences, Okazaki, Japan. The experiments were undertaken in compliance with national legislation and the Code of Ethical Principles for Medical Research Involving Human Subjects of the World Medical Association (Declaration of Helsinki). All participants provided written informed consent.

### Evaluation of Subjective Happiness

The SHS provides a relatively stable measure of whether an individual is a happy or unhappy person (Lyubomirsky and Lepper, 1999). In the SHS, participants were required to rate statements using a seven point Likert scale. The items were as follows: (1) In general, I consider myself: (1 = not a very happy person/7 = a very happy person). (2) Compared with most of my peers, I consider myself: (1 = less happy/7 = more happy). (3) Some people are generally very happy. They enjoy life regardless of what is going on, getting the most out of everything. To what extent does this characterization describe you? (1 = not at all/7 = a great deal). (4) Some people are generally not very happy. Although they are not depressed, they never seem as happy as they might be. To what extent does this characterization describe you? (1 = not at all/7 = a great deal). After reverse coding the fourth item, we computed the mean of the four items to generate a score (Lyubomirsky and Lepper, 1999).

### MRI Data Acquisition

A 3-T scanner (Verio; Siemens, Ltd., Erlangen) was used to acquire data for VBM. Each participant’s head was immobilized within a 32-element phased-array head coil. Whole-brain high-resolution T1-weighted anatomical MRI using a magnetization prepared rapid acquisition gradient echo (MP-RAGE) was conducted on each participant [echo time (TE) = 2.97 ms; repetition time (TR) = 1,800 ms; field of view (FOV) = 256 mm × 256 mm; flip angle = 9°; matrix size = 256 pixels × 256 pixels; and slice thickness = 1 mm).

### MRI Data Analysis

We used VBM8 toolbox revision 435^1^ with SPM8 revision 4667 (Wellcome Trust Centre for Neuroimaging^2^) in MATLAB 2013a (MathWorks, Inc.) to analyze structural images (Ashburner and Friston, 2000; Ashburner, 2007). The images were corrected for bias-field inhomogeneity and spatially normalized with diffeomorphic anatomical registration using exponentiated Lie algebra; tissues were classified as gray matter, white matter, or cerebrospinal fluid (Ashburner and Friston, 2000; Ashburner, 2007). During the modulation process, non-linear deformations were used for normalization, such that the voxel intensities reflected regional gray matter densities adjusted for individual brain sizes. Images were then smoothed to a Gaussian kernel of 8-mm full width at half maximum (FWHM). After preprocessing of the structural images, gray matter densities were submitted to second-level analysis. A two-sample *t*-test, with gender and age as effects of no interest, was used to test for inter-group differences. A region of interest (ROI) analysis approach was adopted, focusing on the striatum (caudate, putamen, and nucleus accumbens) as defined by Wake Forest University PickAtlas^3^. The significance threshold was set at *p* < 0.05 (*t* > 2.765), corrected for family-wise error (FWE) at the voxel level by applying small volume corrections. To check for a possible relationship between SHS score and gray matter density within clusters exhibiting significant differences between the groups, we conducted regression analysis between the SHS score and average beta value within significant clusters for all 68 participants in both groups, and 36 participants in the in-relationship group only. In addition, in an exploratory analysis, we also conducted whole-brain analysis to investigate gray matter density differences between the groups using the same significance threshold [*p* < 0.05 (*t* > 4.844)].

Similar to romantic relationship status, the history of formation of romantic relationships (e.g., duration of being in romantic relationship) may also contribute to modulation of striatal gray matter density. Thus, to evaluate the possible contribution of romantic relationship experience, we also conducted group-level VBM analysis of data from 56 participants (in-relationship group) and performed multiple regression. In the multiple regression analysis, we defined the duration of being in a romantic relationship as an effect of interest, and gender and age as effects of no interest.

## Results

The mean ± SEM of SHS score was 5.1 ± 0.1 in the in-relationship group, and 4.6 ± 0.2 in the no-relationship group. SHS scores differed significantly between the two groups, as determined by *t*-test [*t*(66) = 2.26, *p* = 0.027].

**FIGURE 1:**
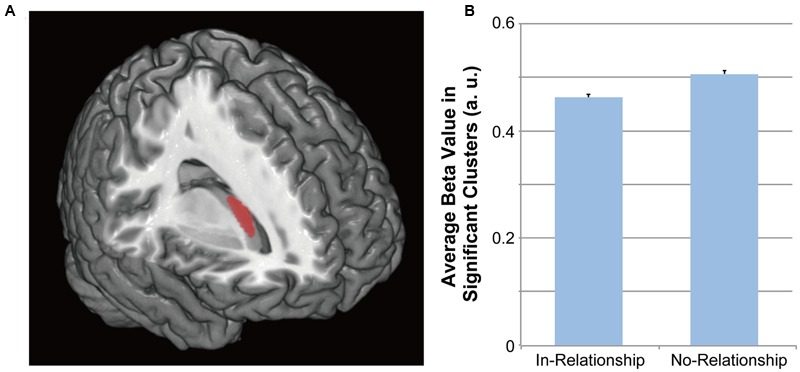
**Differences in gray matter density. (A)** Location of a significant cluster in the right dorsal striatum. The threshold for the gray matter density differences was set at a voxel level of *p* < 0.05 (*t* > 2.765), FWE-corrected with an anatomical striatal mask. **(B)** Average beta values in the cluster related to in-relationship and no-relationship groups.

**FIGURE 2:**
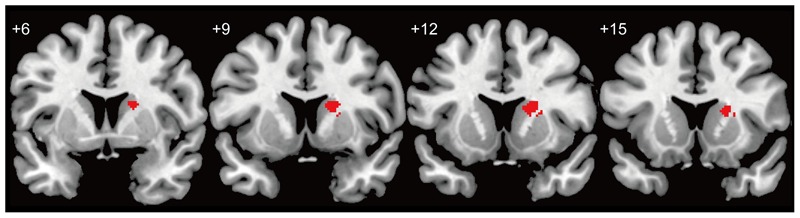
**Cluster location related to being in a romantic love.** Red area indicates differences in gray matter density between groups (no-relationship > in-relationship). The threshold for a difference in gray matter density was set at a voxel level of *p* < 0.05 (*t* > 2.765), FWE-corrected with an anatomical striatal mask.

Voxel-based morphometry results showed that the gray matter density of the right dorsal striatum [top peak = (26, 14, 12)] of the in-relationship group was significantly lower than that of the no-relationship group (**Table 1**; **Figures 1** and **2**). Furthermore, because we did not find any significant correlation between average beta-value in significant clusters and SHS scores [two groups (68 participants): *r*(66) = -0.120, *p* = 0.331; in-relationship group (36 participants): *r*(34) = -0.114, *p* = 0.508], the significant difference in gray matter density in the right dorsal striatum could be primarily attributed to being in a romantic relationship. In the whole-brain analysis, we did not find any significant group differences except in the right dorsal striatum [top peak = (26, 14, 12); FWE-corrected *p* = 0.029; number of voxels = 2].

**Table 1:** Significant difference in gray matter density between groups (no-relationship > in-relationship).

	Peak *p* (FWE)	MNI coordinates			Cluster size	*t*-value
		*x*	*y*	*z*		
Right dorsal striatum	0.001	26	14	12	178	4.99
Right dorsal striatum	0.037	22	9	10	2	3.94

*The threshold of differences related to gray matter density was set at a voxel level of *p* < 0.05 (*t* > 2.765), FWE-corrected with an anatomical striatal mask.*

On the other hand, in terms of VBM results related to the duration of being in a romantic relationship, neither striatal ROI analysis nor whole-brain analysis revealed significant correlations.

## Discussion

### Enhancement of Subjective Happiness Associated with Being in Romantic Love

The in-relationship group had a significantly higher subjective happiness score than the no-relationship group. The in-relationship group also had a higher subjective happiness score than average Japanese participants in a previous study, whereas the no-relationship participants had a lower-than-average score (Shimai et al., 2004; Matsunaga et al., 2011). These results suggest that being in a romantic relationship might enhance subjective happiness, whereas not being in a romantic relationship might decrease it. The quality of a romantic relationship (i.e., relationship satisfaction) enhances subjective happiness (Knee et al., 2005), whereas the subjective evaluation of one’s own wealth does not (Diener and Seligman, 2002); thus, whether or not one is in a romantic relationship might modulate the subjective happiness felt by the participants. Subjective happiness experienced in a romantic relationship depends on the features of the intimate relationship (Hazan and Shaver, 1987), and influences the perception of one’s own existence (Aron et al., 1995; Taylor et al., 2003; Lewandowski et al., 2006). Because early adults typically spend long periods of time with romantic partners (Reis et al., 1993), possibly due to their influential positive nature (Bartels and Zeki, 2000; Aron et al., 2005), the significant difference in subjective happiness between the in-relationship and no-relationship groups suggests that being in an early-stage romantic relationship is accompanied by influential positive experiences, which might in turn lead to structural changes in the brain.

### Gray Matter Density Modulation in the Striatum Associated with Being in a Romantic Relationship

The VBM experiment revealed that early-stage romantic love was associated with a reduction of gray matter density in the striatum. The striatum represents monetary reward (Knutson et al., 2001; Izuma et al., 2008) and social reward, such as the ‘warm glow’ (i.e., the pleasant feeling caused by helping behavior) (Harbaugh et al., 2007), empathic joy (Kawamichi et al., 2013), receipt of active listening (Kawamichi et al., 2015b), social interaction (Kawamichi et al., 2016), and receipt of praise from others (Izuma et al., 2008). Thus, the striatum represents a common neural currency of reward (Izuma et al., 2008), and in this sense is a key node for reward processing (O’Doherty, 2004). Because viewing a romantic partner activates the striatum, the presence of a romantic partner appears to act as a social reward (Bartels and Zeki, 2000, 2004; Aron et al., 2005; Xu et al., 2011; Acevedo et al., 2012). As expected, the results of the VBM experiment suggested that structural differences in the striatum might be related to “social reward” stimuli associated with being in a romantic relationship.

Synaptic plasticity plays a pivotal role in modulation of gray matter density detectable by MRI (Lerch et al., 2011; Zatorre et al., 2012). Synaptic remodeling, which modulates the possibility of neuronal excitation, is a major factor leading to changes in gray matter density (Zatorre et al., 2012). Because gray matter loss detected by MRI is primarily due to loss of dendrites and their synapses (Kassem et al., 2013), the observed reduction in gray matter density might reflect loss of synapses. This type of gray matter modulation can occur in timescales on the order of weeks (e.g., 2 weeks’ abstinence from alcohol) (van Eijk et al., 2013). Because the minimum duration for being in a romantic relationship in this study was 1 month, relationship status could plausibly have modulated gray matter density in the dorsal striatum through synaptic remodeling.

Individual experiences can alter gray matter density within the adult human brain (Zatorre et al., 2012). Progressive reduction in striatal gray matter density has been observed during post-adolescent brain maturation (Sowell et al., 1999). Furthermore, cortico-striatal-thalamic-cortical circuits are influenced by a possible developmental component (Colibazzi et al., 2008). Through spending long periods of time with romantic partners (Reis et al., 1993), experiences related to romantic relationship may contribute to adult development. Thus, influential positive experiences resulting from being in an early-stage romantic relationship may cause a reduction in striatal gray matter density.

Similar to romantic relationship status, the history of involvement of romantic relationships (e.g., duration of being in romantic relationship) could also contribute to modulation of striatal gray matter density. However, neither striatal ROI analysis nor whole brain analysis showed significant correlations. Previous studies showed that the effects of structural brain changes caused by experience-dependent mechanisms are reversible [e.g., sports (Freund et al., 2014), chronic pain (Gwilym et al., 2010), or obsessive-compulsive disorder (Lazaro et al., 2009)]. Dissolution of a romantic relationship has effects opposite to those of forming the relationship. Humans feel social pain as a result of the dissolution of a romantic relationship (Macdonald and Leary, 2005), and dissolution has a negative impact on physical and emotional responses (Kiecolt-Glaser and Newton, 2001; Davis et al., 2003). Furthermore, dissolution harms health (Mearns, 1991; Rhoades et al., 2011). Accompanying these negative effects, dissolution of a romantic relationship has a detrimental impact on self-concept (Lewandowski et al., 2006). Because humans have substitution motivations to form romantic relationships at the time of dissolution (Baumeister and Leary, 1995), individuals can recover from a dissolved romantic relationship by forming another romantic relationship. Thus, being in a romantic relationship might also have a reversible effect. Taking into consideration the results of these previous studies, as well as the results from our exploratory analysis, it would appear that the effect of romantic relationship status on striatal gray matter density modulation is greater than that of the history of being in romantic relationships, possibly due to the reversibility of these effects.

These results imply that the striatum is tuned to respond to reward (Langen et al., 2011) through experiences related to being in an early-stage romantic relationship. In regard to disruption of the tuning function of the striatum, individuals with autism exhibit striatal activation in response to specific for focused interests (Kohls et al., 2013), but not social or monetary reward (Dichter et al., 2012). Furthermore, the reward circuitry in individuals with autism exhibits enlargement of the striatum (Herbert et al., 2003; Langen et al., 2007, 2009). Striatal enlargement is correlated with repetitive behavior (Amaral et al., 2008; Langen et al., 2009), and thus might represent insensitivity to a broad range of reward. By contrast, patients with cocaine dependence or alcoholism have reduced gray matter density in both the dorsal (Barros-Loscertales et al., 2011) and ventral striatum (Makris et al., 2008). The reduction in gray matter density in dorsal striatum of cocaine-dependent patients may be associated with an enhanced response to reward, accompanied by deficits in inhibitory control (Barros-Loscertales et al., 2006). This kind of inhibitory control is mediated by the dopamine D2 receptor, which is also downregulated in drug addiction (Martinez et al., 2004). Therefore, the reduced gray matter density observed in dorsal striatum of participants in early-stage romantic relationship implies that they share some neurobiological mechanisms with patients with drug addiction, at least to some extent, i.e., increased saliency of the target of addiction or romantic partner. Thus, being in a romantic relationship covaries with the enhancement of reward-related responses toward the target (i.e., affection toward the romantic partner) through increased saliency of social cues, reflecting a reduction in gray matter density in the striatum.

Unlike drug addiction, every encounter related to romantic relationships has a strong social component. Such social encounters trigger release of oxytocin and vasopressin in the mesolimbic dopamine pathway (Burkett and Young, 2012). Furthermore, given this social component, social attachment exerts a protective function against addiction (Tops et al., 2014). Given that D2 receptor in the striatum is downregulated in patients with drug addiction (Martinez et al., 2004), and social relationships increases resilience with respect to drug addiction by increasing levels of D2 receptor (Morgan et al., 2002), being in a romantic relationship might exert different effects on D2 receptors. Consistent with this idea, we observed a laterality difference in dorsal striatum reduction: our results showed that being in a romantic relationship was associated with reduced gray matter density in the right dorsal striatum, whereas drug addiction was associated with a reduction in the left dorsal striatum (Barros-Loscertales et al., 2011). This laterality difference fits with reward-related dopamine response in striatum: greater D2 receptor availability in the left striatum is related to incentive motivation (Tomer et al., 2008), which leads to high achievement. Furthermore, this laterality difference is supported by studies showing a correlation between right dorsal striatal activation and strength of affection (Aron et al., 2005) or between reduction in gray matter volume in the right dorsal striatum and the amount of time spent viewing sexual visual images (Kuhn and Gallinat, 2014). An increase in subjective happiness for in-relationship participants might be accompanied with this reward-related response enhancement through romantic relationship–specific effects on the dopamine receptor.

Social support from intimate others, such as romantic partners, helps to alleviate stress and prevent feelings of distress (Mikulincer et al., 2003; Coan et al., 2006), leading to an improved sense of well-being (House et al., 1988). In this kind of stress alleviation mechanism, positive feelings aroused through social support attenuate responses to aversive stimuli (Younger et al., 2010; Kawamichi et al., 2015a). Thus, positive feelings represented as a reduction in gray matter density in the dorsal striatum, might perform a stress-buffering function that promotes the psychological and physical well-being associated with being in a romantic relationship.

On the other hand, we did not observe any significant correlation between subjective happiness and gray matter density in dorsal striatum. Furthermore, a previous study found a significant correlation between subjective happiness and gray matter density in the most ventral part of medial prefrontal cortex (mPFC) (Matsunaga et al., 2016). Similar to the striatum, the most ventral part of mPFC represents social reward (Moll et al., 2006). Specifically, it integrates reward value across different stimuli or stimulus dimensions (Blair et al., 2006) by connecting to striatum (Haber et al., 2006). Information related to being in a romantic relationship increases happiness by integrating with other aspects of social status or relationships (Diener and Seligman, 2002). With this result, even romantic relationship and addiction share some neural mechanisms, romantic relationship has specific effects on subjective happiness. In this sense, being in a romantic relationship, which is an example of a positive relationship, primarily modulates the dorsal striatum with specifically accompanying increment in subjective happiness.

### Limitations

Although the primary target of the present study was the covariation of gray matter density with current romantic relationship status, the aforementioned reversibility of the effects of romantic relationships is another important issue regarding the underlying neural mechanisms. From this perspective, future studies should investigate the detailed mechanisms of this reversibility, in terms of personal experiences related to previous romantic relationship and romantic relationship quality (to investigate the factors responsible for modulation of the reversible effects) or being in love without being in a romantic relationship (to investigate the onset of the reversible effects). Such studies would also provide additional insight pertinent to the results reported here; i.e., they could elucidate whether these factors have effects on gray matter density in the dorsal striatum. Furthermore, future studies should also seek to replicate our results regarding subjective happiness by using a larger sample size, thereby overcoming potential problems related to the discrepancy between the number of participants in the VBM analysis and the subjective happiness analysis.

This study has another limitation; namely, we did not obtain information regarding the causal connection between gray matter density in the dorsal striatum and romantic relationship status. Therefore, it is also possible that reduced gray matter density influences the possibility of having romantic relationships with others. To investigate this causal relationship, it would be necessary to perform a longitudinal study in which MRI was performed before and after subjects entered romantic relationships.

## Conclusion

Being in an early-stage romantic relationship enhanced subjective happiness, suggesting the existence of positive influential experiences associated with such relationships. Furthermore, being in an early-stage romantic relationship was associated with reduced gray matter density in the dorsal striatum. Because the dorsal striatum represents reward expectation, humans in romantic relationships might have increased saliency of social reward (romantic relationship), represented as lower gray matter density in the dorsal striatum, mediated by experiences aroused by being in a romantic relationship in daily life.

## Author Contributions

HK designed the experiments, conducted the experiments, analyzed the data, and wrote the manuscript. SKS helped to conduct the experiments and discussed the data. YHH, KM, and MM helped to conduct the experiments. HCT discussed the data and wrote the manuscript. YO and SS discussed the data. NS supervised the overall project and edited the manuscript.

## Conflict of Interest Statement

The authors declare that the research was conducted in the absence of any commercial or financial relationships that could be construed as a potential conflict of interest.

## Footnotes

1 http://dbm.neuro.uni-jena.de/vbm/

2 http://www.fil.ion.ucl.ac.uk/spm/

3 http://www.fmri.wfubmc.edu/software/PickAtlas/
